# Leaf Traits That Contribute to Differential Ozone Response in Ozone-Tolerant and Sensitive Soybean Genotypes

**DOI:** 10.3390/plants8070235

**Published:** 2019-07-20

**Authors:** Amanda Bailey, Kent Burkey, Matthew Taggart, Thomas Rufty

**Affiliations:** 1 USDA-ARS, Plant Science Research Unit, Raleigh, ND 27695-7631, USA; 2 Crop and Soil Sciences Department, North Carolina State University, Raleigh, ND 27695-7620, USA

**Keywords:** genetic variation, leaf gas exchange, ozone, soybean, stomatal conductance, water-use efficiency

## Abstract

Ozone (O_3_) is a phytotoxic air pollutant that limits crop productivity. Breeding efforts to improve yield under elevated O_3_ conditions will benefit from understanding the mechanisms that contribute to O_3_ tolerance. In this study, leaf gas exchange and antioxidant metabolites were compared in soybean genotypes (Glycine max (L.) Merr) differing in ozone sensitivity. Mandarin (Ottawa) (O_3_-sensitive) and Fiskeby III (O_3_-tolerant) plants grown under charcoal-filtered (CF) air conditions for three weeks were exposed for five days to either CF conditions or 70 ppb O_3_ in continuously stirred tank reactors (CSTRs) in a greenhouse. In the CF controls, stomatal conductance was approximately 36% lower for Fiskeby III relative to Mandarin (Ottawa) while the two genotypes exhibited similar levels of photosynthesis. Ozone exposure induced significant foliar injury on leaves of Mandarin (Ottawa) associated with declines in both stomatal conductance (by 77%) and photosynthesis (by 38%). In contrast, O_3_ exposure resulted in minimal foliar injury on leaves of Fiskeby III with only a small decline in photosynthesis (by 5%), and a further decline in stomatal conductance (by 30%). There was a general trend towards higher ascorbic acid content in leaves of Fiskeby III than in Mandarin (Ottawa) regardless of treatment. The results confirm Fiskeby III to be an O_3_-tolerant genotype and suggest that reduced stomatal conductance contributes to the observed O_3_ tolerance through limiting O_3_ uptake by the plant. Reduced stomatal conductance was associated with enhanced water-use efficiency, providing a potential link between O_3_ tolerance and drought tolerance.

## 1. Introduction

Ozone (O_3_) is a problematic greenhouse gas and air pollutant when present at high levels in the troposphere of the Earth’s atmosphere [1,2,3]. Current levels of O_3_ have significant effects on the growth and yield of many plants, including agricultural crops [2,4,5]. The economic losses caused by O_3_ are estimated at $3 to $5 billion each year, and the losses will continue as O_3_ concentrations increase over time [1,6]. Among the most O_3_-sensitive crops is the soybean, which is also the fourth most economically important crop in the United States [6,7].

The susceptibility to O_3_ damage varies greatly among plant species and genotypes within species [8,9,10]. Genetic variation in O_3_ response in soybean can be seen in the varying levels of decreased biomass production, seed yield, stomatal conductance, and carbon assimilation, as well as increased foliar injury [6,9,11,12,13,14,15]. The variation in soybean genotype O_3_ response provides a useful research opportunity to understand O_3_ tolerance mechanisms. 

Physiological mechanisms that may contribute to genetic variation fall into two general categories: (1) gas exchange processes that limit O_3_ uptake into leaves and (2) metabolic processes that either detoxify O_3_ in the leaf apoplast or alter the cellular signaling pathways that regulate O_3_ response. The first involves exclusion of O_3_ based on differences in the rate of stomatal conductance. As stomatal conductance increases, more O_3_ enters the leaf and reacts with the plasma membrane to produce reactive oxygen species (ROS) that initiate a signaling cascade, which can lead to damage of the leaf [15]. Several studies suggest that the O_3_ exclusion mechanism may contribute to O_3_ tolerance in soybean [16,17], but the results are not always conclusive. These inconsistencies could be due to inherent genotype differences or the confounding effects of leaf developmental stage. In any case, O_3_ exclusion only partially explains O_3_ tolerance because genotypes with similar stomatal conductance can exhibit variation in O_3_ response [3,18]. 

Another contributing factor in the differential response to O_3_ exposure could be variation in antioxidant metabolism among genotypes [11,15]. Antioxidants have the potential to detoxify harmful compounds such as ROS that are produced in response to O_3_ entry into leaves [19]. Ascorbic acid and glutathione are two such antioxidants that are involved in regulating ROS produced by O_3_ and are present at high concentrations in leaves across plant species [20,21]. Ascorbic acid is found in both the apoplast and cytoplasm while glutathione is only in the cytoplasm [22]. In soybeans and snap beans, some studies show that significant differences exist between tolerant and sensitive genotypes for reduced ascorbic acid, total ascorbic acid, reduced glutathione, and oxidized glutathione [17,23]. However, a few studies show no significant difference between tolerant and sensitive genotypes in regard to the ascorbic acid and glutathione content of leaves [16,18]. Further research is needed to characterize the differences in ascorbic acid and glutathione concentrations in soybeans, particularly an assessment of canopy position and leaf age effects that may explain the conflicting reports in the literature.

The present study investigates tolerance mechanisms in soybean to O_3_ by examining the effects of O_3_ exposure on photosynthesis, stomatal conductance, and ascorbic acid and glutathione levels in two genotypes of soybean, Fiskeby III and Mandarin (Ottawa). These genotypes are known to be O_3_-tolerant and sensitive, respectively [9]. Data were collected from three different leaf positions to determine the role of leaf aging and to find out whether the inconsistencies in plant responses to O_3_ are due to variations in leaf age. Understanding genotypic differences in stomatal conductance and antioxidants concentrations could help elucidate the mechanisms of O_3_ tolerance in plants. Understanding the mechanisms of O_3_ tolerance will contribute to breeding efforts to improve crops in the future.

## 2. Results

### 2.1. Ozone Study 

#### 2.1.1. Experimental Conditions

Ozone exposure averages were 5.2 and 65.6 ppb for charcoal-filtered (CF) and O_3_ treatment, respectively, for the first experiment and 5.3 and 68.3 ppb for CF and O_3_ treatment, respectively, for the second experiment. The average relative humidity and temperature during the first experiment’s exposure period was 49% and 37 °C, and 55% and 35 °C in the second experiment exposure period. Average photosynthetically active radiation (PAR) during the exposure periods was 300 and 194 µmol m^−2^ s^−1^ for the first and second experiment, respectively. It should be noted that on the day of gas exchange measurements the ambient PAR for the first experiment was 348 µmol m^−2^ s^−1^ while the second experiment’s PAR was 119 µmol m^−2^ s^−1^. The PAR difference between the two measurement dates was significantly different (*p* = 0.0347, Table A1).

#### 2.1.2. Leaf Injury

There was a significant second-order interaction between leaf age, treatment, and genotype in both experiments (*p* = 0.0035, *p* = 0.0008; Table 1 and Table 2).

**Figure 1:**
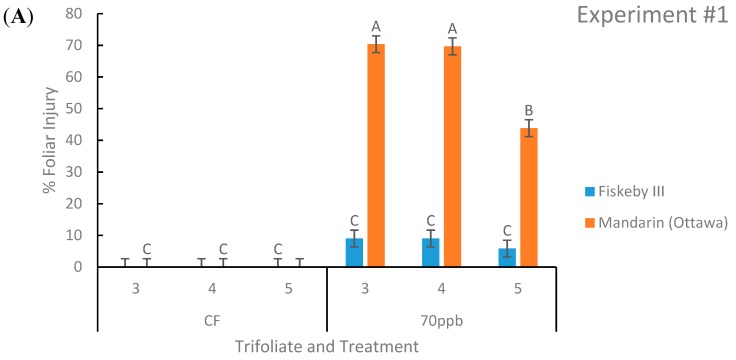

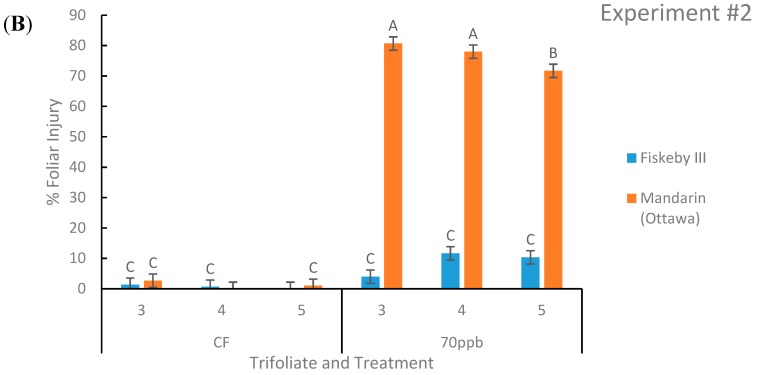
Foliar injury of soybean leaves represented as the percent area of the leaf displaying damage. Two genotypes were exposed to either charcoal-filtered (CF) air or 70 ppb O_3_ for 5 days, 7 h/day. Values are least squared means ± the associated standard error for *n* = 3. Leaf positions for assessment of leaf age effects are the 3rd, 4th, and 5th main stem trifoliate leaves numbered acropetally. Panels (**A**) (Experiment #1) and (**B**) (Experiment #2) represent the two independent experiments conducted sequentially. The letters above the bars are Tukey test results where different letters indicate the least squared means are significantly different.

Ozone-treated Mandarin (Ottawa) had significantly greater leaf injury than Fiskeby III in both experiments at all three leaf positions of interest. Across both experiments Mandarin (Ottawa) had an average of 69% of leaf area damaged by O_3_ compared to Fiskeby III with 9% of leaf area damaged (Figure 1). Fiskeby III did not develop significant foliar symptoms during O_3_ exposure, although a small amount of injury was detected under the exposure conditions employed here (Figure 1). Mandarin (Ottawa) developed significant foliar injury during O_3_ exposure at all leaf positions, the youngest leaf having significantly less damage than the older two leaves.

#### 2.1.3. Leaf Gas Exchange

##### Net Photosynthesis (A)

**Figure 2:**
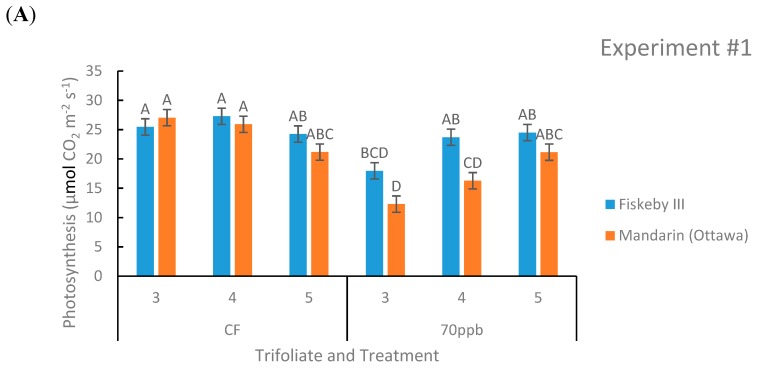

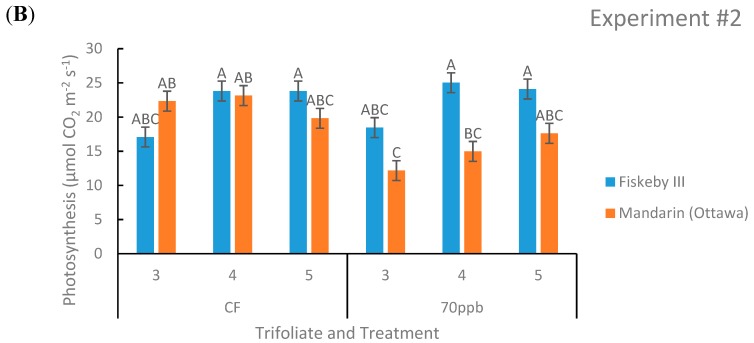
Photosynthetic rate of two soybean cultivars treated with either CF air or 70 ppb O_3_ for 5 days, 7 h/day. Measurements were made at three different leaf positions. Values are least squared means ± the associated standard error for *n* = 3. Leaf positions for assessment of leaf age effects are the 3rd, 4th, and 5th main stem trifoliate leaves numbered acropetally. Panels (**A**) (Experiment #1) and (**B**) (Experiment #2) represent the two independent experiments conducted sequentially. The letters above the bars are Tukey test results where different letters indicate the least squared means are significantly different.

Net photosynthesis was not significantly different between Mandarin (Ottawa) and Fiskeby III in CF treatment at any leaf age tested in both experiments (Figure 2). In both experiments there was a consistent significant genotype by treatment effect (*p* = 0.0021 and *p* = 0.0354). 

Under O_3_ treatment, Fiskeby III had significantly higher rates of A at some leaf positions tested, but overall there was a trend that Fiskeby III had higher rates than Mandarin (Ottawa) (Figure 2). Other parameters yielded significant results but not in both experiments. In Experiment #2 the interaction between treatment, genotype, and leaf age was significant (*p* = 0.0371), but this was not seen in Experiment #1. Experiment #1 also had a significant treatment by leaf age effect (*p* = 0.0003) that was not seen in the other experiment. 

##### Stomatal Conductance (g_s_)

**Figure 3:**
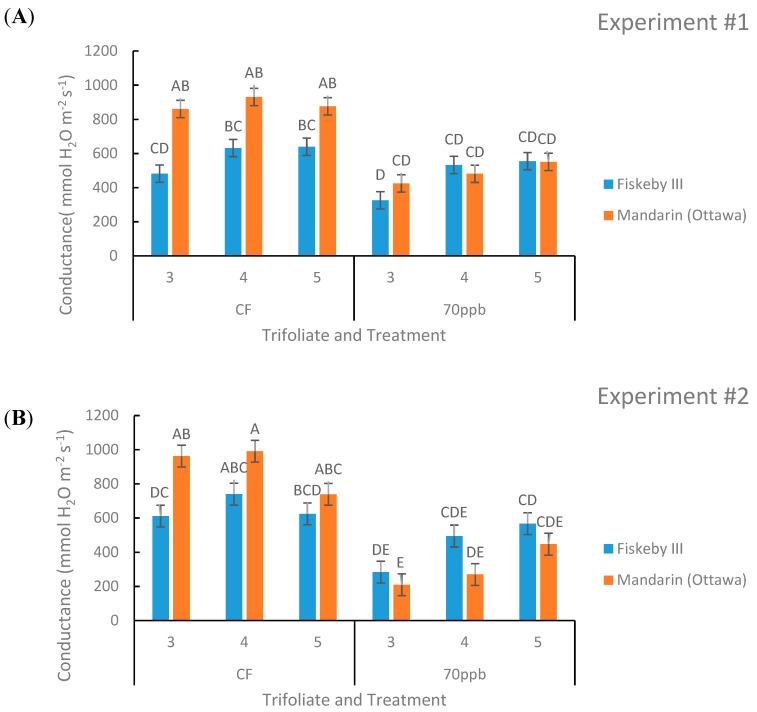
Stomatal conductance (g_s_) of two soybean genotypes exposed to CF or 70 ppb O_3_ for 5 days, 7 h/day. Values are least squared means ± the associated standard error for *n* = 3. Leaf positions for assessment of leaf age effects are the 3rd, 4th, and 5th main stem trifoliate leaves numbered acropetally. Panels (**A**) (Experiment #1) and (**B**) (Experiment #2) represent the two independent experiments conducted sequentially. The letters above the bars are Tukey test results where different letters indicate the least squared means are significantly different.

Differences in stomatal conductance between the two genotypes were observed in Experiment #2 (*p* = 0.0153, Table 2) but not in Experiment #1 (*p* = 0.1591, Table 1). In general, Fiskeby III exhibited lower conductance than Mandarin (Ottawa) (Figure 3).

In the CF treatment there is an obvious trend that Mandarin (Ottawa) has a higher rate of conductance compared to Fiskeby III, although the difference is only significant in the oldest trifoliate measured (Figure 3). The interaction between treatment and genotype had a significant impact on stomatal conductance in both experiments (*p* = 0.0028 and *p* = 0.0209, Table 1 and Table 2). Ozone exposure significantly reduced stomatal conductance in Mandarin (Ottawa) in the two oldest measured leaf positions consistently across experiments, and the trend continued for the youngest leaf (Figure 3). Significant reductions in conductance due to O_3_ exposure were not observed for Fiskeby III at any leaf age (Figure 3). Leaf age had a significant effect on conductance for the second experiment (*p* = 0.0003, Table 2). The interaction between leaf age and treatment had a significant effect during the first experiment (*p* = 0.004, Table 1).

##### Internal CO_2_ (Ci)

**Figure 4:**
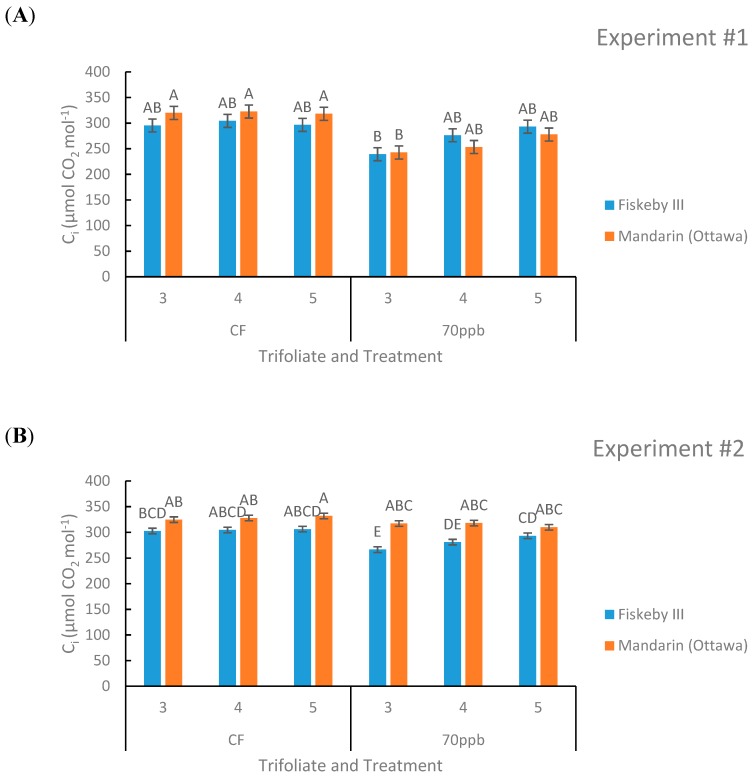
Internal CO_2_ (Ci) concentration of two soybean genotypes exposed to either CF air or 70 ppb O_3_ for 5 days, 7 h/day. Values are least squared means ± the associated standard error for n = 3. Leaf positions for assessment of leaf age effects are the 3rd, 4th, and 5th main stem trifoliate leaves numbered acropetally. Panels (**A**) (Experiment #1) and (**B**) (Experiment #2) represent the two independent experiments conducted sequentially. The letters above the bars are Tukey test results where different letters indicate the least squared means are significantly different.

Effects on Ci were not consistent across both experiments. In Experiment #1 the O_3_ treatment effect reduced Ci (*p* = 0.0317), and there was a trend toward reduction in Ci in the second experiment as well (*p* = 0.0686, Table 1). In Experiment #1 leaf age had a significant effect on Ci (*p* = 0.0377, Table 1), but in Experiment #2 there was only a trend (*p* = 0.0624, Table 2). There was also a significant second-order interaction between treatment, genotype, and leaf age present in the second experiment that was not seen in the first experiment. In the CF treatment there is no significant difference between the Ci values of Mandarin (Ottawa) or Fiskeby III, although there was a trend for greater Ci in Mandarin (Ottawa) (Figure 4). The Ci response to O_3_ did not consistently differ during exposure. In the first experiment there was no difference between Mandarin (Ottawa) and Fiskeby III during O_3_ treatment, in the second experiment Fiskeby III had significantly lower Ci in the leaf cohort measured (Figure 4). 

##### Intrinsic Water-Use Efficiency (WUE) Calculated from Leaf Gas Exchange Measurements

**Figure 5:**
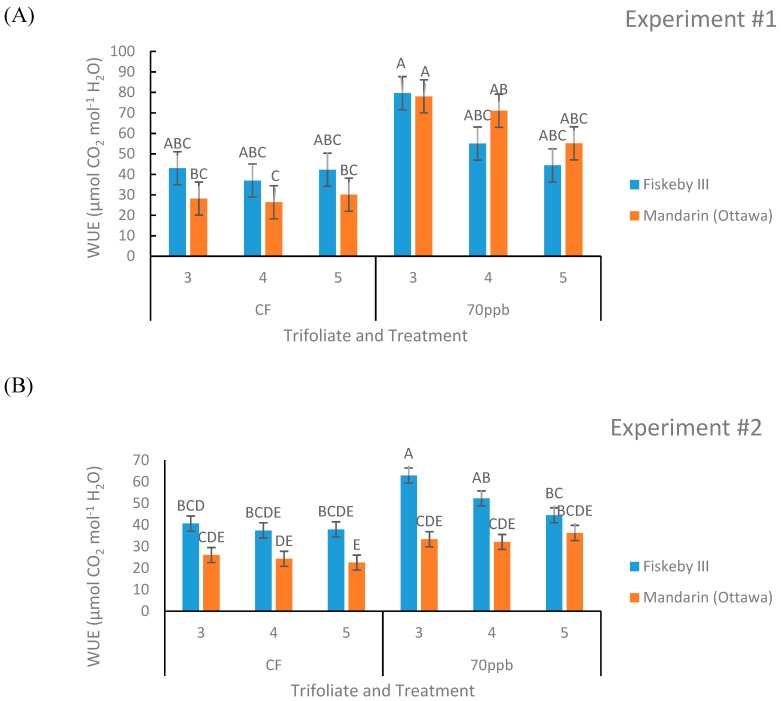
Intrinsic water-use efficiency (WUE) of two soybean genotypes treated with either CF air or 70 ppb O_3_ for 5 days, 7 h/day. Leaf gas exchange measurements taken with the LI-COR 6400 were used to calculate WUE for three different leaf positions. Values are least squared means ± the associated standard error for *n* = 3. Leaf positions for assessment of leaf age effects are the 3rd, 4th, and 5th main stem trifoliate leaves numbered acropetally. Panels (**A**) (Experiment #1) and (**B**) (Experiment #2) represent the two independent experiments conducted sequentially. The letters above the bars are Tukey test results where different letters indicate the least squared means are significantly different.

There is a significant leaf age effect on WUE in both the experiments (*p* = 0.0341, *p* = 0.0242, Table 1 and Table 2) with higher WUE in the older leaves measured. Other effects on WUE were observed but were not consistent across both experiments. Ozone treatment significantly increased WUE in Experiment #1 (*p* = 0.0328) only, and Fiskeby III had significantly higher WUE in Experiment #2 (*p* = 0.0026). The first-order interaction between treatment and trifoliate had a significant effect only in Experiment #1 (*p* = 0.0303, Table 1). The second experiment had a significant interaction between genotype and leaf age (*p* = 0.0442, Table 2) as well as a significant second-order interaction between treatment, genotype, and leaf age (*p* = 0.0268, Table 2). Fiskeby III tended to have a higher WUE than Mandarin (Ottawa) in the CF treatment in both experiments, though this trend is clearest in the second experiment (Figure 5). The WUE of the O_3_-treated plants was not consistent for Mandarin (Ottawa) across the two experiments, however, Fiskeby III tended to have higher WUE under O_3_ stress in both experiments (Figure 5). Although it was not significant, there was a trend of WUE being higher in the O_3_ treatment compared to CF for comparable cultivar and leaf age (Figure 5). 

#### 2.1.4. Ascorbic Acid 

**Figure 6:**
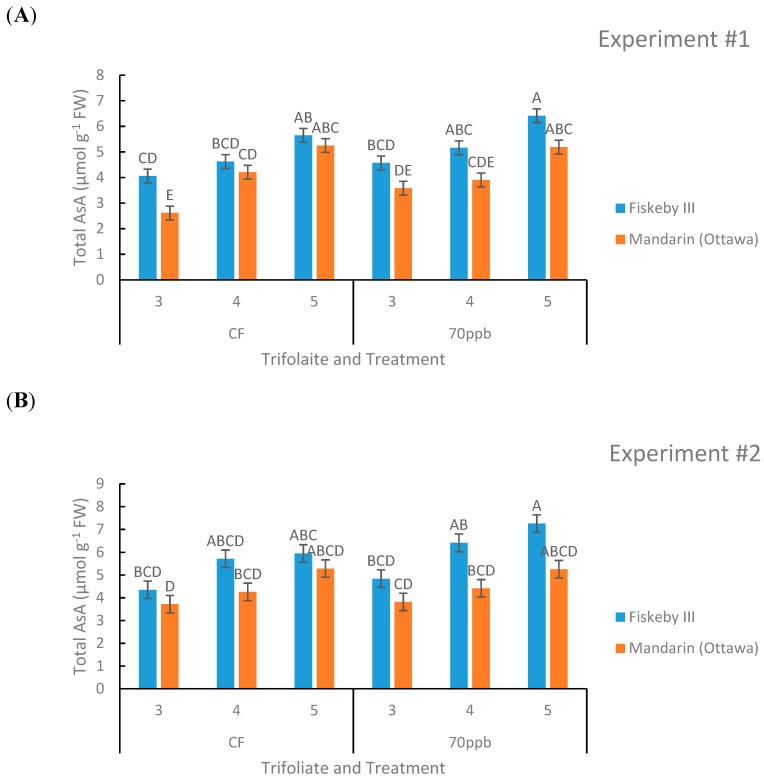
Total ascorbic acid concentration in the leaves of two soybean genotypes treated in either CF air or 70 ppb O_3_ for 5 days, 7 h/day. Values are least squared means ± the associated standard error for *n* = 3. Leaf positions for assessment of leaf age effects are the 3rd, 4th, and 5th main stem trifoliate leaves numbered acropetally. Panels (**A**) (Experiment #1) and (**B**) (Experiment #2) represent the two independent experiments conducted sequentially. The letters above the bars are Tukey test results where different letters indicate the least squared means are significantly different.

Genotype (*p* = 0.0005, *p* = 0.0057, Table 1 and Table 2) and leaf age (*p* = 0.0001, *p* = 0.0001, Table 1 and Table 2) both had a significant effect on total leaf ascorbic acid (AsA) content in both experiments. There was a trend in both treatments and both genotypes that younger leaves contained more AsA than older leaves (Figure 6). The O_3_-treated soybeans had higher levels of AsA compared to their CF-treated counterparts in both genotypes. There was a trend that Fiskeby III had higher leaf AsA than Mandarin (Ottawa) in both treatments and all leaf ages measured, and this was consistent across both experiments (Figure 6). 

#### 2.1.5. Glutathione (GSH)

**Figure 7:**
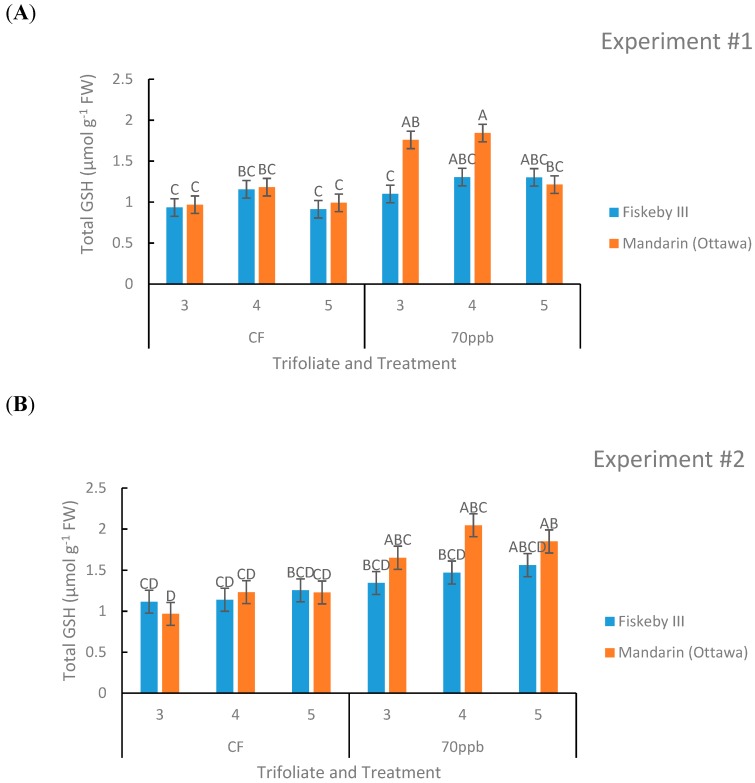
Total glutathione (GSH+GSSG) concentration in the leaves of two soybean genotypes exposed to either CF air or 70 ppb O_3_ for 5 days, 7 h/day. Values are least squared means ± the associated standard error for *n* = 3. Leaf positions for assessment of leaf age effects are the 3rd, 4th, and 5th main stem trifoliate leaves numbered acropetally. Panels (**A**) (Experiment #1) and (**B**) (Experiment #2) represent the two independent experiments conducted sequentially. The letters above the bars are Tukey test results where different letters indicate the least squared means are significantly different.

Leaf age had a significant effect on glutathione levels in both experiments (*p* = 0.0001, *p* = 0.0107) with higher glutathione content in Trifoliate #4 representing the leaf of cohort of intermediate age (Figure 7). Treatment effect was significant in the second experiment (*p* = 0.0312) with a trend in the first experiment (*p* = 0.0715) toward increasing leaf glutathione content of O_3_ treated plants. The first-order interaction between genotype and trifoliate was significant in the first experiment and so was the second-order interaction between treatment, leaf age, and genotype (*p* = 0.0001). In the second experiment there was a significant treatment by leaf age interaction (*p* = 0.0107).

In the CF treatment there were no significant differences between the two genotypes or trends that appeared. In the O_3_-treated plants there was a trend of Mandarin (Ottawa) containing more glutathione than Fiskeby III, especially in older leaves. There was also a trend of glutathione content increasing due to O_3_ exposure, especially in Mandarin (Ottawa) where the differences were sometimes significant (Figure 7).

#### 2.1.6. Ascorbic Acid and Glutathione Redox Status

Reduced forms of ascorbic acid and glutathione predominated in soybean leaves with little variation found across genotypes or O_3_ treatments. The only significant effect was on ascorbic acid redox status in the second experiment (*p* = 0.0238), where the percent of AsA in the reduced form was highest in the 2nd youngest leaf measured. No other effects or interactions were significant for either antioxidant metabolite (Table 1 and Table 2). Across all combinations of genotype and treatment, 92% of AsA (range 84–99%) and 96% of glutathione (range 93–99%) were found in the reduced form.

### 2.2. Response of Whole-Plant Transpiration to Vapor Pressure Deficit

**Figure 8:**
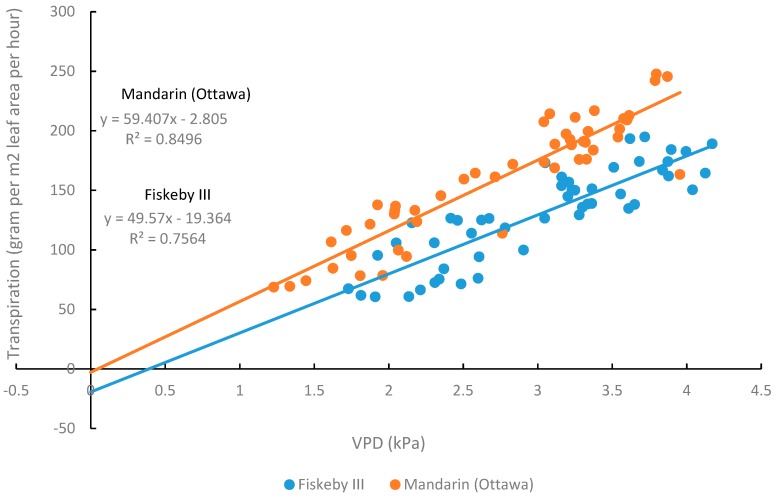
The transpiration rate of two soybean genotypes at different vapor pressure deficit (VPD) levels. Lines of linear regression are for each genotype individually.

For both genotypes, the whole-plant transpiration rate increased with increasing VPD (Figure 8). Fiskeby III generally had lower whole-plant transpiration rates than Mandarin (Ottawa) with the difference becoming more evident at higher VPD levels. The slope of the lines representing the relationship between whole-plant transpiration and VPD were marginally different between the two genotypes while the intercepts of the two lines were significantly different (Table 3). 

## 3. Discussion

Photosynthesis is a known target of O_3_ effects [15]. In this study, O_3_ exposure inhibited photosynthesis by an average of 38% in O_3_-sensitive Mandarin (Ottawa) for main stem leaves exhibiting leaf injury scores of 40–82%. Photosynthesis declined, but only by 6%, in O_3_-tolerant Fiskeby III where injury scores ranged from 1–15%. This confirms that foliar symptoms are associated with loss of photosynthetic capacity. 

While both genotypes exhibited similar rates of photosynthesis in the absence of O_3_ stress, differences in stomatal conductance were observed (Table 1 and Table 2). In the CF controls, stomatal conductance of Mandarin (Ottawa) averaged 36% higher than Fiskeby III across the two experiments with differences greatest in the oldest leaf cohort (Figure 3). The consistently lower rates of stomatal conductance in Fiskeby III supported a role for stomatal exclusion as a factor contributing to the reduced O_3_ sensitivity. These results supported previous work from our lab where a trend toward higher conductance in Mandarin (Ottawa) compared with Fiskeby III was found in a study that evaluated an older leaf cohort [16]. In an independent study of different germplasm, O_3_ sensitivity was associated with higher stomatal conductance in two Amazonian soybean genotypes [17]. Thus, lower stomatal conductance is a physiological trait associated with O_3_-tolerance that results from reduced O_3_ uptake into leaves and less foliar damage. The mechanism that supports high rates of photosynthesis at lower stomatal conductance in Fiskeby III remains unknown. Genotype differences in Rubisco content and/or activation are possible controlling factors [24].

In general, O_3_ exposure results in reduced stomatal conductance [11], and this was observed in this study as well. Our results showed a significant interaction between genotype and treatment on stomatal conductance across both experiments (Figure 3). Exposure of Mandarin (Ottawa) to elevated O_3_ was associated with a 77% reduction in stomatal conductance compared to a 30% reduction in Fiskeby III averaged across all leaf cohorts in both experiments. In an independent study of other soybean cultivars, there was a significant decrease in stomatal conductance following O_3_ exposure in both O_3_-sensitive Forrest and tolerant Essex genotypes [18], evidence that stomatal exclusion is only one of several possible tolerance mechanisms present in soybean germplasm. Overall, O_3_ reduces stomatal conductance in soybean, but the severity of the reduction is variable and depends upon genotype and the environmental conditions during exposure. 

Stomatal exclusion mechanisms potentially impact WUE, the rate of carbon fixation in relation to the rate of water loss in plants [25]. The inherently lower stomatal conductance of Fiskeby III (Figure 3) and similar rates of photosynthesis (Figure 2) compared with Mandarin (Ottawa) resulted in a greater intrinsic WUE for Fiskeby III (Figure 5). This was most clearly observed under CF conditions where results were not confounded by O_3_ effects on leaf gas exchange. WUE also increased for both genotypes with O_3_ exposure as the result of greater impacts on stomatal conductance than on photosynthesis. This differs from a field study where WUE, based on seed yield and calculated evapotranspiration, declined in response to O_3_ exposure [26]. These differences reflect differences in WUE measurement approach or possible differences in cultivar response. 

The differences in stomatal conductance between Fiskeby III and Mandarin (Ottawa) at the leaf level using the LI-COR 6400 gas exchange system were also apparent at the whole-plant level using a gravimetric approach to observe genotype differences in whole-plant transpiration response to variable vapor pressure deficit (VPD). Transpiration and stomatal conductance are closely related processes where a lower rate of transpiration is indicative of a lower rate of stomatal conductance and greater WUE. Typically, as VPD increases, the rate of transpiration also increases, and this was observed for Fiskeby III and Mandarin (Ottawa) (Figure 8). Genotype differences in the slopes and intercepts of regression lines between whole-plant transpiration and VPD were apparent (Table 3, Figure 8) with Fiskeby III exhibiting a lower rate of transpiration than Mandarin (Ottawa) regardless of the VPD. This suggests that rates of leaf stomatal conductance in Fiskeby III are lower across a wide range of VPD conditions, representing a unique leaf trait. The lower rates of stomatal conductance and transpiration in Fiskeby III suggest a potential link between O_3_ tolerance and drought tolerance. There is genetic evidence to support this linkage. A mapping population developed from a genetic cross between Fiskeby III and Mandarin (Ottawa) has been used to identify a molecular marker on chromosome 6 of Fiskeby III (044133-08626) that is associated with both ozone tolerance [27] and the slow wilting trait used as an indicator for drought tolerance [28].

WUE is an important trait of drought tolerant crop plants but is a very difficult trait to select for in breeding programs [29]. In soybean, one reported drought tolerance trait involves a “break” in the transpiration-VPD curve where the rate of transpiration plateaus at a moderate VPD to conserve soil moisture for reproductive growth later in the season [30]. The enhanced WUE trait in Fiskeby III appears to be a different mechanism where transpiration is lower at all VPD levels. Further research is needed to understand the relationship between WUE and these two VPD traits in soybean. If WUE and O_3_ tolerance are indeed linked through a leaf gas exchange trait, it might be possible for breeding programs to simultaneously select for tolerance to both stresses. 

Of the antioxidant processes evaluated in this study, only genetic variation in leaf AsA content was observed. Fiskeby III AsA levels were 23% higher than Mandarin (Ottawa) when averaged across treatments in the two experiments. No significant genotype differences in glutathione content or the redox status of either the ascorbic acid or glutathione pools were found. It is difficult to interpret the significance of elevated AsA as a contributor to the greater O_3_ tolerance of Fiskeby III. Potential mechanisms include direct detoxification of O_3_-generated ROS by AsA in the leaf apoplast or alteration of redox signaling pathways that regulate O_3_-induced injury responses. Although apoplast AsA content was not measured in this study, a majority of AsA was found in the oxidized form in a previous study of other O_3_-sensisitive and tolerant soybean genotypes [18], suggesting direct apoplast detoxification may not be a factor in soybean. The more complex explanation involves altered redox signaling. ROS formed from O_3_ breakdown in the cell wall is one of many sources of ROS formation that must be regulated in various cellular compartments as part of normal metabolism in plants [20,22]. We cannot know whether the proposed redox control of O_3_ signaling in Fiskeby III might occur at the initial site of ROS formation in the cell wall or through regulation of the ROS signaling between neighboring cells that if uncontrolled leads to lesion formation. 

Leaf age clearly influenced O_3_ responses. Older leaves exposed to O_3_ accumulated greater injury than the younger leaves higher in the canopy (Figure 1). This pattern has been observed previously with other soybean cultivars [18] and other plant species [31]. Leaf age had a significant effect on both AsA and glutathione in both experiments (Table 1 and Table 2). Younger leaves had higher concentrations of AsA, consistent with another study [32]. In contrast, glutathione concentration was highest in the middle leaf cohort. These observations demonstrate the importance of considering leaf developmental stage when evaluating ozone effects on plants. 

In conclusion, we found that stomatal conductance contributes to the differential O_3_ response in Fiskeby III and Mandarin (Ottawa) soybean. Ozone-tolerant Fiskeby III inherently has a lower stomatal conductance, effectively reducing the amount of O_3_ uptake through the stomata while maintaining similar rates of photosynthesis as O_3_-sensitive Mandarin (Ottawa). Fiskeby III also has greater leaf ascorbic acid content that may contribute to greater tolerance. The results suggest that both stomatal exclusion and antioxidant metabolism are factors contributing to the greater O_3_ tolerance of Fiskeby III. We also found that Fiskeby III has a higher WUE than Mandarin (Ottawa) as a consequence of lower stomatal conductance, and this finding was independently confirmed in experiments showing lower whole-plant transpiration in Fiskeby III across a wide range of VPD conditions. The linkage between stomatal exclusion of O_3_ and WUE suggests that this unusual leaf gas exchange trait in Fiskeby III may provide tolerance to both O_3_ and drought. This presents the possibility that breeding for this trait could enhance crop tolerance to both stresses. 

## 4. Materials and Methods 

### 4.1. Ozone Study

#### 4.1.1. Plant Material and O_3_ Treatments

This experiment was conducted twice in a greenhouse in Raleigh, North Carolina, in June 2016. The experiment was a split-split plot with a completely randomized block design. The chamber was the whole plot, O_3_ treatment was the whole plot factor, genotype was the split-plot, and the split-split plot was the main stem trifoliate. There were 3 blocks made of 2 chambers each with a charcoal filter (CF) and O_3_ treatment. Two plants of both genotypes were grown in each chamber. Mandarin (Ottawa) (PI 548379) and Fiskeby III (PI 438471), maturity group 00, were grown in pots with 2 RSI Farfard (Sungro, Agawam, MA, USA) and 15.3 grams of slow release fertilizer (Osmocote Plus, Scotts-Sierra, Maryville, OH, USA) in a greenhouse with charcoal-filtered air for 3 weeks under 20-hour days (04:00 to 24:00) to delay flowering. Vegetative stages averaged V-6.8 for Experiment #1 and V-7.8 for Experiment #2. Two plants of each genotype were placed in six 1.2 m^3^ continuous stir reactor tanks (CSTRs) for a 2-day period of acclimation followed by a 5-day period of treatment. The treatments were either CF or O_3_ for 7 h∙day^−1^ (0900 to 1600). Ozone was generated by a TG-20 Ozone Generator (Ozone Solutions, Hull, IA, USA), and the O_3_ concentrations were monitored by a Model 49C Ozone Analyzer (Thermo Environmental Instruments, Franklin, MA, USA). Relative humidity (RH) and temperature were recorded in each CSTR with El-USB-2LCD (EasyLog, DATAQ Instruments, Akron, OH, USA) every 5 min. Photosynthetically active radiation (PAR) was recorded in 1 CSTR per block with Li-190 Quantum Sensor (LI-COR, Lincoln, NE, USA). 

#### 4.1.2. Foliar Injury

Foliar injury was recorded at all main stem leaf positions on each plant 3 days after the end of the exposure. Injury was determined by estimating the percentage of leaf area that displayed visible injury, such as necrotic or chlorotic lesions and stippling on the leaf surface [18]. Five observers scored the injury, and the results were averaged for each leaf position on each plant for statistical analysis. 

#### 4.1.3. Leaf Gas Exchange

Stomatal conductance and photosynthesis measurements were made on the 4th day of exposure using a LI-COR 6400 (LI-COR, Lincoln, NE, USA). Net photosynthesis, stomatal conductance, and internal leaf CO_2_ were measured at the 3rd, 4th, and 5th trifoliate acropetally on the middle leaflet. Intrinsic water-use efficiency (WUE) was calculated for each trifoliate leaf by dividing net photosynthesis by the stomatal conductance. Measurements from plants of the same genotype in each chamber were averaged together for statistical analysis. Measurements were taken between the hours of 10:00 to 14:00 Eastern Standard Time. The cuvette had an area of 6 cm^2^ and was placed on one side of the mid-vein. PAR was set at 1500 (µmol m^−2^ s^−1^), the relative humidity (RH) was maintained at 55%, and the sample CO_2_ targeted 390 (µmol CO_2_ mol^−1^). The flow was controlled between 300–600 (µmol s^−1^).

#### 4.1.4. Sample Preparation

For ascorbic acid and glutathione assays, the middle leaflet that was measured during gas exchange was collected and placed in liquid nitrogen and transferred to a −80 °C freezer. Leaf tissue for each sample was ground into powder using a mortar and pestle pre-chilled in liquid nitrogen. A known quantity of frozen powder was weighed in a pre-chilled polypropylene centrifuge tube placed on a balance using a pre-chilled spatula and funnel. Ice cold extraction buffer (6% (*w/v*) meta-phosphoric acid, 0.2 mM DTPA) (10 ml g^−1^ FW) was added to centrifuge tube, and the slurry was stirred occasionally until thawed. The tubes containing samples were then centrifuged at 20,000 × *g* for 10 min at 4 °C. 

#### 4.1.5. Ascorbic Acid Assay 

After centrifugation, the supernatant was recovered and assayed for reduced ascorbic acid (AsA) and the oxidized form, dehydroascorbic acid (DHA). The AsA and DHA present in the leaf tissue were determined using the protocol described in Luwe and Heber [33], with the following modifications. The buffer used was 100 mM potassium phosphate (KPi) at pH 7.0, and 25 µL of leaf extract was assayed in 975 µL of KPi. Determination of the AsA concentration was based on complete oxidation of AsA to DHA by ascorbate oxidase and monitoring the change in A265nm. The oxidized form, DHA, was determined by reducing DHA to AA with DTT and monitoring the change in A_265nm_. Total AsA (ascorbic acid in both forms) was calculated as the sum of the reduced and oxidized forms.

#### 4.1.6. Glutathione Assay

After centrifugation, the supernatant was recovered and assayed for determining the total glutathione (GSH + GSSG) present in both reduced (GSH) and oxidized (GSSG) forms from the protocol published by Tietze [34] and oxidized glutathione (GSSG) according to the protocol by Griffith [35]. Both assays are based on a glutathione reductase (GR) recycling reaction leading to the reduction of Ellman reagent (DTNB) to form a colored product (2-nitro-5-thiobenzoic acid) measured at 412 nm with spectrophotometer. A standard curve was created each day to relate known concentrations of GSH to the rate of DTNB reduction. The background rate of GR reduction of DTNB in the absence of GSH was subtracted from all other rates for the construction of the standard curve and determination of unknowns.

#### 4.1.7. Statistical Analysis

The two experiments were analyzed using PROC GLIMMIX in SAS 9.4 (SAS Inc., Cary, NC, USA). They were analyzed using the split-split plot analysis of variance with model:

yijkl = μ + Bi +Tj +Wij + Ck +Sijk+Pl +(T *C)jk +(C*P)kl + (T*P)jl + (C*T*P)jkl + eijkl.

Bi = random block effect

Tj = treatment effect

Pl = leaf position

Wij = random whole plot effect

Ck = cultivar effect

Sijk = random split plot effect

(T*C)jk = treatment by cultivar effect

(C*P)kl = cultivar by leaf position effect

(T*P)jl = treatment by leaf position effect

(C*T*P)jkl = cultivar by treatment by leaf position effect

Eijkl = split-split plot error

The 2 plants of the same genotype in each chamber were averaged together for a chamber mean. Averages were separated according to Tukey’s post-hoc test. 

A similar analysis was conducted on the environmental data for PAR to determine if the two experiments’ growing conditions were significantly different from each other. Since they were significantly different and the days that we did gas exchange measurements were very different, we treated the two experiments separately (Table A1).

### 4.2. Vapor Pressure Deficit Study

#### 4.2.1. Plant Material and VPD Treatments

This experiment was conducted at the North Carolina State University’s Phytotron in Raleigh, NC. Two genotypes of soybean, Fiskeby III and Mandarin (Ottawa), were grown in 5-liter pots filled with 2 RSI Farfard (Sungro, Agawam, MA, USA) and 7.8 grams of slow release fertilizer. Plants were grown for approximately 3 weeks in a phytotron chamber under the conditions of 20-h days, 30/26 °C for day/night temperatures, and approximately 60% RH. 

#### 4.2.2. Water Loss Measurements

The water loss measurements were completed using the general protocol set out by Rosas-Anderson [36]. Pots were wrapped in plastic bags and tightened around the stem of the plant to prevent transpiration from the soil. Ten pots of each genotype were randomly placed on individual balances (Model SP-6001, Scout Pro, readability 0.1 g, Ohaus Corp., Parsippany, NJ, USA) that communicated with a computer outside of the chamber. A range of VPD increments between 1.5–4 kPA were targeted by adjusting ultrasonic humidifiers (model V5100NS, Vicks P&G, Cincinnati, OH) and condensing dehumidifiers (Hisense, Suwanee, GA) to reach the desired RH while the temperature in the chamber was held constant. Plants had a 30-min acclimation period once the next VPD increment was achieved. At each increment the mass of the pot was weighed every minute for 1 h to determine the rate of transpiration for each plant. Each pot had an EL-USB-2-LCD + data logger (Lascar Electronics Ltd., Erie, PA, USA) that recorded temperature and RH. VPD was then calculated on a per pot basis. Leaf area was calculated by harvesting the leaves on each plant and using a LI-3100 (LI-COR, Lincoln, NE, USA) to measure the leaf area. 

#### 4.2.3. Statistical Analysis

Linear regression was used to determine the R^2^ value for Mandarin (Ottawa) and Fiskeby III separately. An analysis of covariance (ANCOVA) was used to determine the difference between the slopes and the intercepts of the regression lines for Mandarin (Ottawa) and Fiskeby III. Genotype was used as the factor with two levels, VPD was the independent variable, and transpiration was the dependent variable. The interaction between VPD and genotype was used to determine the level of significance for the slopes, and the genotype effect was used for the intercepts. Analyses were completed using SAS 9.4 (SAS Institute Inc., Cary, NC, USA).

## Figures and Tables

**Table 1:** Analysis of variance results (*P* values) for Experiment #1. Effects were tested for foliar injury, photosynthetic rate (A), stomatal conductance (g_s_), intrinsic water-use efficiency (WUE), internal CO_2_ (Ci), total ascorbic acid (AsA), total glutathione (GSH), the redox status of ascorbic acid (AsA), and the redox status of glutathione (GSH). *P* ≤ 0.05 is significant.

Effect	Foliar Injury	A	g_s_	Ci	WUE	Total AsA	Total GSH	Redox Status AsA	Redox Status GSH
Ozone treatment (Trt)	0.0054	0.009	0.0098	0.0317	0.0328	0.3008	0.0715	0.7739	0.4770
Genotype	<0.0001	0.0005	0.1591	0.648	0.7562	0.0005	0.1106	0.4124	0.9909
Trt*Genotype	<0.0001	0.0021	0.0028	0.1708	0.1679	0.0974	0.1868	0.7588	0.7860
Trifoilate	0.0004	0.0641	0.1122	0.0377	0.0341	<0.0001	<0.0001	0.7454	0.3882
Trt*Trifoliate	0.0004	0.0003	0.0040	0.0374	0.0303	0.2278	0.0413	0.4761	0.1033
Genotype*Trifoliate	0.0035	0.5648	0.3192	0.5863	0.5543	0.4489	<0.0001	0.3535	0.7543
Trt*Genotype*Trifoliate	0.0035	0.2507	0.4526	0.8038	0.7940	0.1322	<0.0001	0.6165	0.1214

**Table 2:** Analysis of variance results (*P* values) for Experiment #2. Effects were tested for foliar injury, photosynthetic rate (A), stomatal conductance (g_s_), intrinsic water-use efficiency (WUE), internal CO_2_ (Ci), total ascorbic acid (AsA), total glutathione (GSH), the redox status of ascorbic acid (AsA), and the redox status of glutathione (GSH). *P* ≤ 0.05 is significant.

Effect	Foliar Injury	A	g_s_	Ci	WUE	Total AsA	Total GSH	Redox Status AsA	Redox Status GSH
Ozone treatment (Trt)	0.0032	0.1460	0.0389	0.0686	0.0714	0.2176	0.0312	0.0737	0.6686
Genotype	<0.0001	0.0415	0.0153	0.0016	0.0026	0.0057	0.0626	0.9459	0.4352
Trt*Genotype	<0.0001	0.0354	0.0209	0.2259	0.3831	0.1904	0.0422	0.8102	0.2543
Trifoilate	0.0796	0.0002	0.0003	0.0624	0.0242	<0.0001	0.0107	0.0238	0.0002
Trt*Trifoliate	0.0284	0.1317	0.2485	0.6591	0.4359	0.8247	0.6345	0.5167	0.8673
Genotype*Trifoliate	0.0003	0.0150	0.0693	0.0679	0.0442	0.2925	0.1682	0.0832	0.8852
Trt*Genotype*Trifoliate	0.0008	0.0371	0.6053	0.0201	0.0268	0.6657	0.8007	0.7252	0.1357

**Table 3:** Linear regression equations representing the relationship between VPD and whole-plant transpiration. There was not a significant difference in the slope, but there was a significant difference in the intercept.

Mandarin (Ottawa)		Fiskeby III		*P* values	
Equation	R^2^	Equation	R^2^	Slope	Intercept
y = 59.407x − 2.805	0.8496.	y = 49.57x − 19.364	0.7564	0.0734	<0.0001

## Appendix A

**Table A1:** ANOVA table for PAR data from both experiments to determine if the two experiments were significantly different. Data were collected using the ambient light sensor of the LI-COR 6400 gas exchange instrument.

Type III Tests of Fixed Effects				
Effect	Num DF	Den DF	F Value	Pr > F
**date**	1	4	9.90	0.0347
**treatment**	1	4	0.42	0.5509
**date*treatment**	1	4	0.04	0.8426
**genotype**	1	8	1.25	0.2960
**date*genotype**	1	8	0.78	0.4023
**treatment*genotype**	1	8	0.43	0.5296
**date*treatme*genotyp**	1	8	1.08	0.3301
**trifoliate**	2	32	3.25	0.0520
**date*trifoliate**	2	32	0.18	0.8399
**treatment*trifoliate**	2	32	0.22	0.8075
**date*treatme*trifoli**	2	32	0.59	0.5603
**genotype*trifoliate**	2	32	0.56	0.5786
**date*genotyp*trifoli**	2	32	0.32	0.7307
**treatm*genoty*trifol**	2	32	0.72	0.4923
**date*trea*geno*trifo**	2	32	0.64	0.5319
